# National Brain Councils – common challenges and regional cooperation

**DOI:** 10.3325/cmj.2019.60.385

**Published:** 2019-08

**Authors:** Pavle R. Andjus

**Affiliations:** Serbian Brain Council, Belgrade, Serbia *pandjus@bio.bg.ac.rs*

The European Brain Council (EBC), a non-profit organization based in Brussels, is a network of scientific societies, patient organizations, professional societies, and industry partners promoting brain research toward the improvement of lives of patients with brain conditions, mental and neurological alike. Over 20 countries have formed, or are in the process of forming, national brain councils (NBCs). These are independent and multidisciplinary scientific organizations aligned with the main goals of the EBC, which can become EBC members with observer status. Moreover, recently established NBC liaisons to the EBC Board (2 representatives and 2 substitutes from different NBCs) create an active channel that can convey a unifying voice of the NBC network to the EBC and *vice versa*.

The first unifying action of 17 NBCs resulted in initiating the Survey on the Current State of Care for Patients with Brain Diseases at the 4th NBC Academy meeting in Lisbon in April 2018. The survey analysis resulted in “Ten Priorities for National Brain and Mental Health Plans” (1). Previously, the NBC network also supported the position paper and further actions of the EBC to expand brain research in the coming years of the new Framework program (“Horizon Europe 2020”) (2).

In the same vein, on the occasion of the first Regional NBCs meeting (organized by the Serbian Brain Council as a satellite to the FENS Regional Meeting 2019 in Belgrade), we carried out a survey on common challenges and regional cooperation. This was an important first-time attempt to closely identify partners in the region and open venues for collaboration, both regionally and with the EBC. The Survey comprised the following questions:

1. What is in your view the rationale for launching or sustaining a NBC and/or national neuroscience society?

2. What were in your view the most significant challenges that your NBC/neuroscience society has faced during the last 5 years? And to what extent has your NBC/neuroscience society been able to overcome them?

3. What would you consider to be the most significant achievement of your NBC/neuroscience society?

4. Could regional cooperation between NBCs/neuroscience societies benefit the work of your organization? And if yes, could you shortly elaborate on how this could be beneficial?

5. How should the European Brain Council (EBC) support NBCs/neuroscience societies and collaborate with them?

The responders were representatives from six councils/societies: Austrian Neuroscience Association (ANA), Croatian Brain Council (CBC), Hungarian Brain Council (HBC), Hellenic Society for Neuroscience (HSfN), Serbian Brain Council (SrBC), and Turkish Neurological Society (TNS). The outcome of their responses, after the consultations with the Belgrade regional NBCs forum, are presented below.

## Rationale for launching a NBC

Almost all the societies agreed that the main rationale for launching or sustaining an NBC and/or national neuroscience society is to bring together basic neuroscientists with clinicians, patients, pharmaceutical industry, and stakeholders, and to create a national network. The representatives of the majority of NBCs agreed that one of the goals of NBCs should be to increase the knowledge on brain disorders and neuroscience achievements in this field within the general population. Furthermore, the role of NBCs should be to inform patients, their support organizations, and general public about the new scientific discoveries and the progress in treating their diseases. Several NBCs pointed out that NBCs and neuroscience societies should actively promote the international dimension and establish sustainable liaisons within its network and with the EBC. Having this in mind, it seems that regional NBCs would gladly accept a more active regional exchange of views and ideas. It has been stressed that one of the goals of NBCs should be to attract more funding for basic and clinical neuroscience. These efforts should be directed not only at national and European funding agencies but also at stakeholders from the private sector and pharmaceutical industry. One of the important lines of NBCs' activity is to advocate for better health care support for diagnosis and treatment of brain disorders (neurology, psychiatry, neurosurgery, etc).

### Challenges

It is essential to maintain better and more transparent relations with the stakeholders in the ministries and to efficiently recruit and mobilize the membership. Nevertheless, the sustained activity of the NBC Board and EBC support can keep the yearly plans afloat. The regional societies also face the challenge of brain drain, which can be compensated by devoted personal work of active society/council members.

The crucial challenge is the state of health and care for patients, while on the other hand some patient societies are rather small and in need of support. Hence, the message put forward is that an NBC should support the societal value and visibility of patient societies.

An essential challenge shared by the wide NBCs network is defragmentation and decentralization of societies linked to brain research. Actually, one of the main tasks of a Brain Council is to be an umbrella organization representing and unifying all the national neuroscience and psychiatry organizations. This may require additional funding, and some colleagues (eg, CBC) could gain small but still independent funding in order to succeed in this task.

An inter-professional challenge was also revealed, emphasizing “multi-disciplinary conflicts” regarding certain brain related disorders (TNS). At the same time, it is proposed that the councils build close relationships with health ministries and health sector agencies, and organize specific nationwide scientific working groups for individual neurological disease categories (TNS). In addition, there is increasing competitiveness in research, with members simultaneously expecting immediate personal reward. The solution is a pledge to the government to furnish a national research agency with a reasonable budget (ANA). The latter is surely a message accepted by all NBCs, however, ways of achievements are not always budget-related.

### Achievements

While the representatives of NBCs and national neuroscience societies shared similar views on previous topics, the responses regarding achievements were highly heterogeneous.

With the EBC, NBCs became part of a bigger, unifying platform, beyond the national borders. It was emphasized that NBCs should not be “another self-sufficient association” (ANA), ie, they should promote an international partnership, but also produce results – for patients, research, and the general public. In fact, ANA and HBC stressed research-related activities as the main achievements. In Hungary, this was accomplished through a National Brain Research Program. Among the main accomplishments were also meetings and networking events. Thus, TNS emphasized the importance of the Networking Symposium World Brain Year (2014), which increased the awareness for the need of research, establishment of new collaborations, and support for young researchers, while SrBC highlighted their initiative to host the first Regional NBC Meeting during the FENS regional meeting 2019 (FRM2019) in Belgrade.

Finally, the important annual goal of NBCs has been and will continue to be the Brain Awareness Week – a unifying, huge platform of different profiles of experts, experienced persons, students, media representatives, and general population, which is continually increasing the general public awareness of brain functioning and brain disorders across Europe (as stressed by HSfN).

### Benefits

The goals of NBCs would highly benefit from regional cooperation, which enables the participating members to share their experience and help each other in solving local problems. Such internationalization of activities may as well strengthen NBC’s national influence.

Joint efforts are helpful in overcoming mutual challenges, facilitating research activities by greater exposure to funding opportunities, and making treaties with other European countries. Although a large number of meetings could take place in separate countries, it is better to have joint meetings and share solutions to local problems. In fact, ANA cooperates with regional societies for many years. The Annual National Congress of the TNS, on the other hand, always includes a session called “In the Region” and is looking forward to host an EBC representative. HBC recommended building a stronger collaboration between regional NBCs and the Danube Symposium of Neurological Sciences, as a good opportunity to be more influential in this region. HSfN, in cooperation with the Serbian Neuroscience Society and the Israel Society for Neuroscience, organized a FRM regional meeting in 2015. Finally, the FRM2019 held in Belgrade involved the Serbian Neuroscience Society, Romanian Neuroscience Society, and Turkish Society for Neuroscience, while the SrBC organized the regional NBCs Meeting as a satellite conference.

### EBC support

According to the Survey respondents, the EBC should support national brain councils and relevant participating organizations through several different lines of action:

*Guidance in advocacy and outreach.* The EBC has designed a toolbox on this topic, which will hopefully be updated and further developed. It was also suggested that the EBC should (re)activate the institution of “EBC ambassadors,” which could influence relevant stakeholders and improve the visibility of NBCs. The EBC should also help in organizing networking meetings with stakeholders in different countries. The EBC's announcements, action plans, and bulletins are important and could give leads to the necessary steps and actions of an NBC.

*Networking*. The EBC could be instrumental in the internationalization of NBC's efforts and activities, both research and clinical. It is expected to award fellowships for participation at meetings, as well as webex or other web-based connections for clinicians, researchers, or patient organizations. The EBC could participate, and provide speakers, at relevant national congresses, supporting local NBC memberships and networking.

*Mutual projects and fund raising.* The EBC is expected to provide access and support network for mutual funding opportunities. More specifically, financial and other support is sought for the launching of new NBCs. In the longer term, there is a need for the access to more funding opportunities for specific activities or administrative issues. As mentioned above, joint efforts are helpful in overcoming mutual challenges and facilitating common research activities, thus leading to greater exposure to funding opportunities. The EBC is already helping in this sense.

By sending simple but important messages, the EBC could improve the collaboration with NBCs and help in their main mission development, funding of basic and clinical neuroscience, and education of the population on brain disorders through TV, radio, or other media.

In conclusion, the survey offered a few mutual key points relevant for the regional NBC network. It was emphasized that the NBCs’ main goal would be to build a multidisciplinary platform to promote neurology and psychiatry. The link to the EBC is of special importance since it provides the necessary internationalization for the regional exchange of views. In spite of diverse challenges, the Region can demonstrate considerable achievements, such as the National Brain Research Program, different research-related activities, and outreach events. In the same vein, regional benefits are expressed as opportunities for sharing experiences and solutions. Finally, a great deal of expectations is directed toward the EBC support in terms of guidance on how to access mutual funding sources.

## References

[1] Morris RG, Oertel W, Gaebel W, Goodwin GM, Little A, Montellano P (2016). Consensus Statement on European Brain Research: the need to expand brain research in Europe - 2015.. Eur J Neurosci.

[2] Mauguiere F, Trejo JL, Andjus P, Vergara C, Pochet R (2019). Ten priorities for national brain and mental health plans.. Croat Med J.

